# How Do Older Adults Process Icons in Visual Search Tasks? The Combined Effects of Icon Type and Cognitive Aging

**DOI:** 10.3390/ijerph19084525

**Published:** 2022-04-08

**Authors:** Jianfeng Wu, Dongfang Jiao, Chunfu Lu, Chengmin Li, Xiaofang Huang, Suzan Weng

**Affiliations:** 1 Industrial Design and Research Institute, Zhejiang University of Technology, Hangzhou 310023, China; jianfw@126.com; 2 School of Design and Architecture, Zhejiang University of Technology, Hangzhou 310023, China; dongfangjiao@outlook.com (D.J.); zanzae@163.com (S.W.); 3 School of Arts, Zhengzhou Technology and Business University, Zhengzhou 451400, China; chengmin517@126.com; 4 College of Art and Communication, China Jiliang University, Hangzhou 310018, China; huangxiaofang@cjlu.edu.cn

**Keywords:** icon type, cognitive aging, eye tracking, interface design, user performance, visual search

## Abstract

Considering the differences in cognitive aging among older adults, this study examined how older adults process different types of graphic icons in visual search tasks. Fifty-four medical-related icons, including flat icons (FIs), FIs plus text (FIs + text), skeuomorphic icons (SIs), and SIs plus text (SIs + text), were created. The participants were divided into two groups—cognitively normal (CN) and mild cognitive impairment (MCI)—to complete a visual search task. According to the eye-tracking data of the participants, the search performance of the CN group was significantly better than that of the MCI group. In terms of icon types, all older adults performed better at searching for the combinations of icon and text, especially SI + text, which showed the smallest difference in the search performance between the MCI and CN groups. All older adults performed poorly when searching for FIs. The findings of this study considered the differences in cognitive aging among older adults and provided a useful reference for the icon and interface design of graphical user interfaces.

## 1. Introduction

With the growth of the aging population, more and more technologies are used in our daily lives to help improve the quality of life for older adults. As a medium of interaction between technology and users, the graphical user interface (GUI) has also received increased research attention. The selective degradation of vision in older adults can cause a declining performance in interacting with the interface with age. In addition, the encoding speed of the image language of older adults is slower, especially when they have to name images, which is more challenging for older adults than for young people [1,2].

However, previous studies mainly focused on older adults with normal aging. Cognitive decline, such as decline in memory, attention, perceptual speed, or spatial ability, is a part of normal aging [3]. Mild cognitive impairment (MCI) is a transitive state between normal aging and dementia [4,5,6], with worsening verbal memory, visual space, and executive abilities being common [5,7]. Thus, elderly people with MCI are more prone to errors in understanding and searching for icons [8,9,10,11]. Considering the differences in the degree of cognitive aging between older adults with normal aging and older adults with MCI, it is necessary to reexamine GUI design.

Icons are small images representing objects or commands in a GUI and are thus an important visual element of a GUI [12]. As a channel for interpretation between users and information systems, icons are extensively used in human–computer interaction interfaces and play an important role in targeting application or command searching [13,14]. Icon design is transitioning from skeuomorphism to flatness [15,16]. However, there is no consensus on their respective advantages. Skeuomorphic icons (SIs) rely on features such as textures, shadows, and highlights to mimic real-world objects. Some scholars believe that SIs require less mental effort to process and are more suitable for older adults with cognitive impairments [17], while flat icons (FIs) eschew real-world elements by removing the three-dimensional (3D) design elements, unifying graphic design, and using simpler colors [16,18,19]. FIs are more popular among young people [18,20]. 

Regarding the effect of icon types on the search behavior of older adults, several recent studies focused on icon characteristics, such as semantic distance, visual complexity, and familiarity, and how they influence the search performance of older adults [21,22,23,24]. Studies have revealed that icons with high visual complexity impair the search performance of older adults (longer reaction times and more errors) [24], whereas icons with closer semantic distance reduce these effects [21]. On the other hand, researchers have investigated the influence of icon types on the visual search behavior in older adults during visual search tasks, and the results showed that the types of icons affect the search performance and eye movement behavior of older adults [15,18,20]. For example, Chen found that older adults searched for SIs more quickly and accurately than they did FIs [15]. Moreover, the findings of another comparative study in Chinese older adults indicated that the older adults had better visual search performance for SIs than for FIs, showing a shorter search duration and higher accuracy rate [15], indicating that the icons more specifically reflected the metaphorical semantics of everyday life and helped improve the performance of older users [10]. However, another study suggested that the more complex coding dimensions of SIs reduced the usability of such icons, requiring the longest fixation duration and resulting in the lowest search efficiency [25]. Meanwhile, researchers have revealed an association between text labels of icons and users’ visual behavior. Icons with text labels are searched faster than icons or text alone [26]. Recent studies showed that adding semantically appropriate text labels to icons improved older adults’ understanding and recognition of icons [27,28]. However, other studies also showed that text labels put more stress on the limited visuospatial processing resources of the older adults [1,2].

The above research still has some limitations: It is unclear what type of icons improve the search performance of older adults and whether the combination of an icon and text label is beneficial to older adults. From the perspective of research subjects, the existing studies have paid less attention to the differences in cognitive aging within the older population, and whether the search performance for different types of icons varies among older adults with varying degrees of cognitive aging remains inconclusive.

This study examined the following three research topics: (1) the impact(s) of icon types on the visual search of older adults, and under which icon types older adults would perform better; (2) how the degree of cognitive aging of older adults would affect their visual search; and (3) whether the type(s) of icons and the degree of cognitive aging of older adults had a mutual influence on the search performance of older adults, particularly which type(s) of icons reduced the difference in visual search performance caused by the degree of cognitive aging. Based on the visual search paradigm, this study used eye-tracking technology to capture the visual activities of older adults when they searched for icons. Combined with the behavioral performance, we further illustrated the impact(s) of icon types and cognitive aging on the performance of the older adults in icon searching. The results of this study may help GUI designers in interface development and in user behavior research.

## 2. Materials and Methods

### 2.1. Experimental Design

This study adopted a 4 × 2 between-group design and selected 2 factors as independent variables: (1) icon type, i.e., FI, SI, FI plus text (FI + text), and SI plus text (SI + text); and (2) cognitive level: cognitively normal (CN) and MCI. Icon type was a within-subject variable, and cognitive level was a between-subjects variable. The dependent variables used in this study were as follows: (1) search accuracy—the proportion of the target icons being hit correctly; (2) reaction time (s)—the time from the presentation of the experimental material to the correct selection of the target icon; (3) time to first fixation (TFF; s)—the time from the presentation of the experimental material to the participant’s first gaze at an area of interest (AOI), i.e., time required for the participant to search for the target icon, which reflected the effective search efficiency; (4) fixation duration (FD; s)—the total time the participant’s gaze fixed on the AOI; the longer the fixation duration, the more difficulties in cognitive processing of the target icon. In this study, the AOI was the area of the target icon in a visual search task.

### 2.2. Selection and Design of the Experimental Materials

Studies have shown that the healthcare system usage of older adults is affected by GUI design and icon design [29,30]. Hence, this study selected icons related to healthcare systems as the experimental materials. All icons related to medical services from the hospital medical system websites and medical public self-service terminal interfaces in Beijing, Shanghai, and Hangzhou were selected by a focus group composed of 3 experienced GUI designers as references to create 118 icons and to name all icons with the best text labels to represent the meaning of the icons. Each text label corresponded to an FI and an SI. There were 54 FIs and 54 SIs in total.

Previous studies have shown that the usability of icons is affected by user familiarity and complexity [31,32]. The semantic difference of icons is reduced with increasing familiarity and experience [33,34,35]. Since one of the aims of this study was to investigate which icon type(s) could shorten the visual search performance of MCI and CN, a total of 20 older adults who were cognitively normal as assessed by the Montreal Cognitive Assessment (MoCA) were recruited as volunteers before the formal experiments. These 20 older volunteers were required to rate each icon on a 7-point scale for complexity, familiarity, and semantic distance. The male-to-female ratio of these participants was 1:1, with age ranging from 60 to 70 years (mean ± standard deviation: 61.0 ± 1.84 years). The MoCA is a universal screening tool for cognitive impairment and has been shown to be highly effective [36,37].

Complexity was defined as the icon having abundant and complex details (Score 1 = very simple, Score 7 = very complex). Familiarity was defined as the participant already being familiar with the icon (Score 1 = very unfamiliar, Score 7 = very familiar). Close semantic distance was defined as accurate if the name was considered to describe the icon accurately (Score 1 = very inaccurate, Score 7 = very accurate). According to the scoring results, 27 FIs and SIs with the same text label content were screened out, and one-way analysis of variance (ANOVA) was performed to ensure that the selected 54 icons all had the same complexity, familiarity, and semantic distance. The results showed no significant differences in complexity [F(1, 52) = 2.78, *p* = 0.101], familiarity [F(1, 52) = 3.53, *p* = 0.066], or semantic distance [F(1, 52) = 2.28, *p* = 0.137) between the FI and SI groups. Table 1 shows the mean scores and standard deviations of icon complexity, familiarity, and semantic distances in detail.

**Figure 1:**
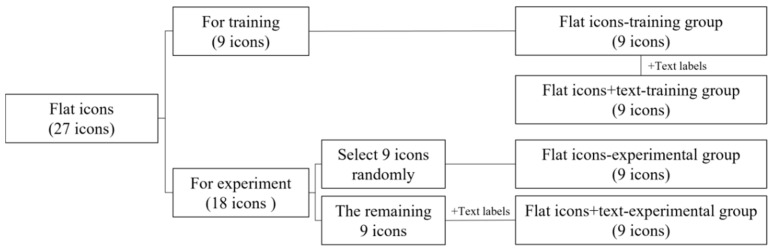
Flowchart showing the grouping of experimental materials.

Figure 1 is a flowchart showing the experimental material grouping. Taking FIs as an example, 27 icons were randomly divided into 2 groups, including 9 icons for training and 18 icons for the experiment. The 9 icons for training formed the FI-training group. Text labels were added to these 9 icons to form the FI + text-training group. The 18 icons used for the experiment were randomly divided into 2 groups (*n* = 9 each). To avoid the learning effect in the experiment, 1 group was selected to add text labels to form the FI + text-experimental group, and the other group without text labels formed the FI-experimental group. The SIs were similarly divided into different groups, namely the SI-training group, SI + text-training group, SI-experimental group, and the SI + text-experimental group.

**Figure 2:**
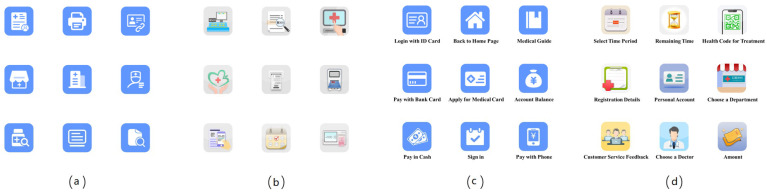
Examples of different groups of experimental materials. (**a**) Flat icons (FIs); (**b**) skeuomorphic icons (SIs); (**c**) FIs plus text; (**d**) SIs plus text.

In each icon group, 1 icon was randomly selected as the target, and the remaining 8 were distractors, arranged in a 3 × 3 manner to form stimulus material 1 of the group in the experiment. Since a cross located in the center of the screen was used in eye-tracking experiments to fix the gaze point of the participant before each task, we avoided placing the target icon in the middle of the screen. Then, the above steps were repeated without using the previous target until 5 experimental stimuli were generated for the group. The above operations were performed for each icon group. A total of 40 stimulus materials were generated among the 4 icon types—FI, SI, FI + text, and SI + text—of which 20 were used for practicing and the remaining 20 were used for formal experiments. Figure 2 shows all stimulus materials presented on a white background.

### 2.3. Experimental Equipment and Participants

#### 2.3.1. Experimental System

The Tobii T60 Eye Tracker and Tobii Studio were used for the presentation of experimental stimuli and data acquisition. The Tobii T60 Eye Tracker is a one-piece telemetry device with a sampling rate of 50 Hz, a resolution of 1280 × 1024 pixels, an accuracy of 0.4° of viewpoint position, and a precision of 0.03°. The binocular data acquisition was adopted, and the head movement range of the participant was 44 cm × 22 cm × 30 cm. Experimental stimuli were displayed on a 17-inch LCD monitor with a resolution of 1920 × 1080 pixels. The participants were tested in a natural sitting position, and the distance between the participant’s eyes and the screen was approximately 65–70 cm.

#### 2.3.2. Participants

Participants were recruited from several communities in Hangzhou, China, with people over the age of 60 years randomly contacted by phone calls from community workers. More participants were subsequently recruited by snowball sampling. A total of 16 participants were eventually included in this study. All participants had normal or corrected-to-normal vision and were in good health for the week prior to the experiment. The results of MOCA were used to divide the participants into the CN (with a score of 26) and MCI (with a score < 26) groups. The research protocol of this study was approved by the Ethic Committee of the Institute of Industrial Design of Zhejiang University of Technology (Zhejiang Province, China). All participants read and signed a consent form before participating in the experiment and received a certain amount of experimental remuneration after the experiment ended.

The 16 participants ranged in age from 60 to 66 years old (mean ± standard deviation = 61.69 ± 1.78 years), including 8 people in the cognitive normal (CN) group (4 males and 4 females, mean ± standard deviation = 62.13 ± 2.1 years) and 8 people in the MCI group (4 males and 4 females, mean ± standard deviation = 61.25 ± 1.39 years). All participants had at least one experience in using self-service check-in machines in medical public terminals (or other public terminals) in the last 3 months and had received 8.44 ± 2.13 years of education. Table 2 shows the demographic information of the participants in this study.

### 2.4. Experimental Design and Procedures

**Figure 3:**
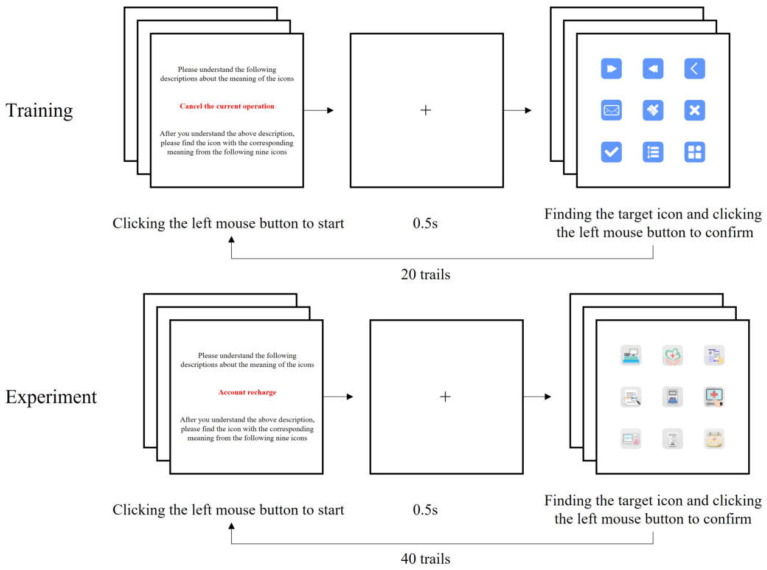
Schematic diagram of the experimental procedures.

All experiments were carried out in the laboratory. After participants filled out the consent form, the pre-test experimental instructions were carried out. The task was to search for and confirm the target icon, and Figure 3 shows the experimental procedures of this study.

Participants were required to first read a text description of the target icon and click the left mouse button to go to the next page after understanding the description. Each test began with gazing at the cross for 0.5 s, and subsequently, the screen automatically switched to a 3 × 3 array of icons. Participants were required to select an icon that matched the target meaning and to click the left mouse button to confirm their selection. The screen would remain the same until the mouse was clicked. After clicking, the next task was presented until all tasks were completed. Participants were given a 5 min break after the training session before a formal experiment in which they were asked to perform the same icon-searching task similar as in the training session. All experimental stimuli were randomly assigned to the participants, and a Latin square design was used to minimize learning effects. The behavior of all participants was recorded by the experimental system.

### 2.5. Statistical Analysis

In this study, the collected data were analyzed using repeated measures ANOVA to examine the single effect of within-subject factors and between-subjects factors for icon types (i.e., FI, SI, FI + text, and SI + text) and cognitive levels (i.e., CN and MCI) of the participants on visual search accuracy, response time, TFF, and FD and their interactions. The SPSS 22.0 software package (IBM, Armonk, NY, USA) was used for statistical analysis. All F statistics passed Mauchly’s sphericity test, using an alpha of 0.05 to test significance in this study.

## 3. Results

A male participant (64 years old) in the CN group was excluded from data analysis due to interruption of eye tracking due to a wide range of head movement during the experiment. The means (standard deviations) of search accuracy, response time, TFF, and FD for the remaining 15 participants are shown in Table 3. Table 4 shows the ANOVA results for icon types and cognitive levels.

### 3.1. Visual Search Accuracy

For all participants, the effect of different icon types on visual search accuracy was significant (*p* < 0.001), with a pairwise comparison of different icon types indicating the following descending order: SI + text, FI + text, SI, and FI. The highest visual search accuracy was for SI + text, which was significantly higher than that for SI and FI (both *p* < 0.001), but the difference from that for FI + text was not significant (*p* = 0.772). FI + text had the second highest accuracy, with an accuracy higher than SI (*p* = 0.001) and FI (*p* < 0.001); SI was less accurate than FI + text (*p* = 0.001) and higher than FI (*p* < 0.001). FI had the lowest accuracy (*p* < 0.001 compared to all other icon types). In addition, the varying cognitive levels among the participants had a significant impact on the visual search accuracy (*p* = 0.017), and the visual search accuracy of the CN group was higher than that of the MCI group. However, the interaction effect of icon type × cognitive level on visual search accuracy was not significant (*p* = 0.118).

### 3.2. Search Response Time

Different icon types had a significant impact on the response time of all participants (*p* < 0.001), and a pairwise comparison of different icon types indicated the following ascending (short to long) order: SI + text, FI + text, SI, and FI. The search response time of SI + text was the shortest, significantly shorter than that of SI and FI (both *p* < 0.001), but the difference from that of FI + text was not significant (*p* = 0.099); the FI + text search response time was shorter time than that of SI (*p* = 0.003) and FI (*p* < 0.001); the SI search response time was longer than that of FI + text (*p* = 0.003) but shorter than that of FI (*p* < 0.001); the search response time for FI was the longest (*p* < 0.001 compared to all other icon types). In addition, the effect of the search response of different cognitive levels was significant: the response time of the CN group was shorter than that of the MCI group (*p* < 0.001).

**Figure 4:**
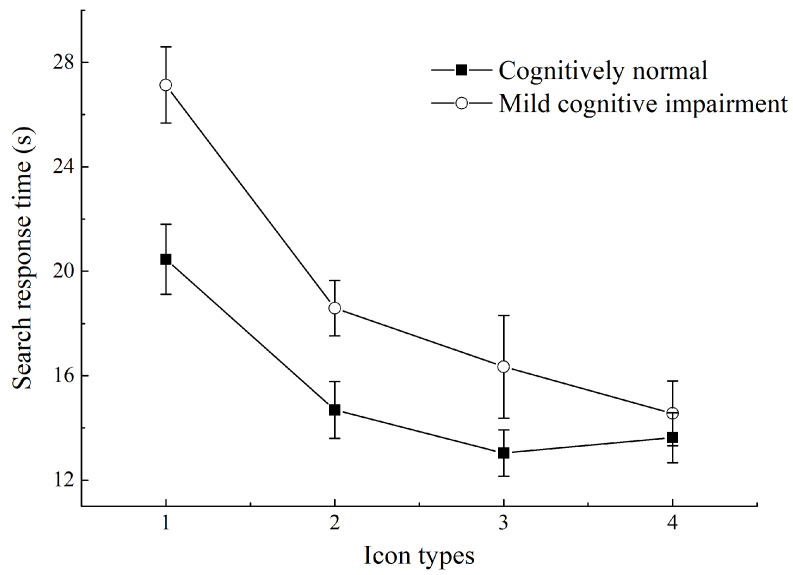
Search response time for different icon types in older adults with different cognitive levels. (1) Flat icons (FIs); (2) skeuomorphic icons (SIs); (3) FIs plus text; (4) SIs plus text.

The interaction effect of icon type × cognitive level on response time was significant (*p* = 0.006). Figure 4 shows the search response time of experiments on the visual search task of FI, SI, FI + text, and SK + text. Further analysis revealed that the icon types had significant effects on both CN (*p* = 0.002) and MCI (*p* < 0.001). However, the icon types with the shortest search response time differed between the CN and MCI groups. The CN group had the shortest search response time for FI + text and had a similar response time for SI + text (*p* = 0.248). The MCI group had the shortest search response time for SI + text, which was significantly faster than the search response time for FI + text (*p* = 0.002). In addition, the two groups showed no significant difference in the search response time for SI + text (*p* = 0.133). Both the CN and the MCI group had the longest search response time for FIs (*p* < 0.001, compared to all other icon types), while the CN group had a significantly faster response time for FI than the MCI group (*p* = 0.008). 

### 3.3. Time to First Fixation (TFF)

Among all participants, different icon types had a significant impact on TFF (*p* < 0.001), and a pairwise comparison of different icon types indicated the following ascending (short to long) order: SI + text, FI + text, SI, and FI. The shortest TFF was that of SI + text, which was significantly shorter than that of SI and FI (both *p* < 0.001), but the difference from that of FI + text was not significant (*p* = 0.145); the TFF of SI is shorter than that of SI + text and FI + text but significantly longer than that of FI (both *p* < 0.001); the longest TFF was for FI (*p* < 0.001 compared to other icon types). The effect of different cognitive levels of participants on TFF was also significant. The CN group had significantly shorter TFF than the MCI group (*p* < 0.001).

**Figure 5:**
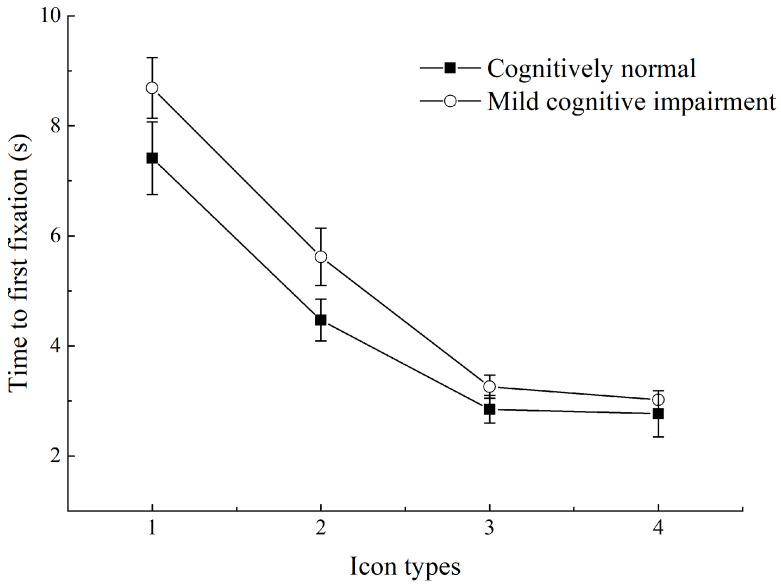
Time to first fixation for different icon types in older adults with different cognitive levels. (1) Flat icons (FIs); (2) skeuomorphic icons (SIs); (3) FIs plus text; (4) SIs plus text.

The interaction effect of icon type × cognitive level on TFF was significant (*p* = 0.003). Figure 5 shows the TFF for the search task of FI, SI, FI + text, and SK + text. Further analysis revealed that icon type had a significant effect on both CN (*p* < 0.001) and MCI (*p* < 0.001). The TFF for different icon types is listed in the order of short to long as follows: SI + text, FI + text, SI, and FI. Participants were able to rapidly locate the SI + text, with no significant differences in TFF between the CN and MCI groups (*p* = 0.152). In addition, the TFF was similar for SI + text and FI + text in both groups (CN: *p* = 0.673, MCI: *p* = 0.103). All groups had the longest TFF for FIs (compared with any other icon type, *p* < 0.001 for all). Nevertheless, the CN group had shorter TFF for FIs than the MCI group (*p* = 0.001).

### 3.4. Fixation Duration (FD)

Among all participants, different icon types had a significant impact on the FD, and a pairwise comparison of different icon types indicated the following ascending (short to long) order as follows: SI + text, FI + text, SI, and FI. The shortest FD was that of SI + text (*p* < 0.05 compared to all other icon types), followed by that of FI + text (*p* < 0.001); SI had a shorter FD than SI + text and FI + text and a longer one than FI (*p* < 0.001); FI had the longest FD (*p* < 0.001 compared to other icon types). The effect of different cognitive levels of participants on FD was also significant. The CN group had a significantly shorter FD than the MCI group (*p* < 0.001).

**Figure 6:**
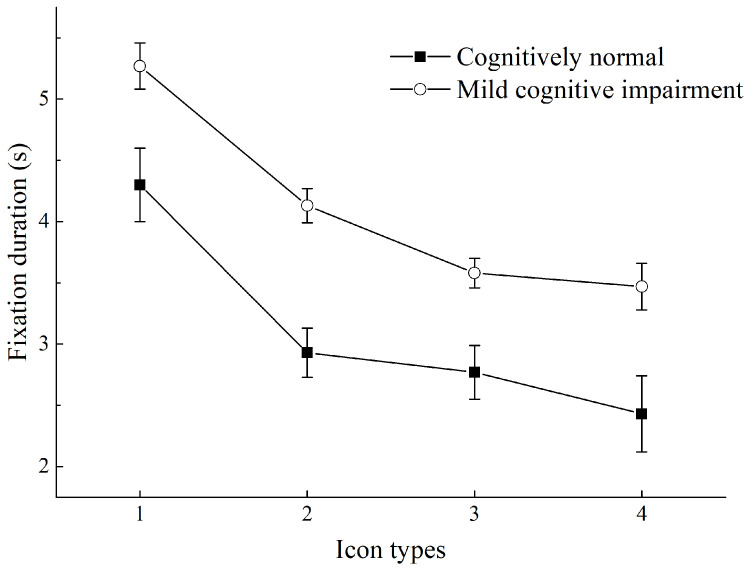
Fixation duration for different icon types in older adults with different cognitive levels. (1) Flat icons (FIs); (2) skeuomorphic icons (SIs); (3) FIs plus text; (4) SIs plus text.

The interaction of icon type × cognitive level had a marginally significant effect on the FD (*p* = 0.053). Figure 6 shows the FD for the search task of FI, SI, FI + text, and SK + text. Further analysis revealed that icon type had a significant effect on both CN (*p* < 0.001) and MCI (*p* < 0.001). SI + text had the shortest FD, followed by FI + text, SI, and FI. All participants had the shortest FD for SI + text. However, the CN group had a shorter FD for SI + text than the MCI group (*p* < 0.001). The FD for SI + text and that for FI-text were not significantly different in the MCI group (*p* = 0.180) but were significantly different in the CN group (*p* = 0.001). All participants had the longest FD for FI icons, (*p* < 0.001, compared with all other icon types); nevertheless, the CN group had a significantly shorter FD in FIs than the MCI group (*p* < 0.001).

## 4. Discussion

### 4.1. Main Findings

#### 4.1.1. Effect of Icon Types on Visual Search Performance of Older Adults

This study showed that among the four types of icons, i.e., FI, SI, FI + text, and SI + text, all the older adults had the best visual search, processing, search response time, and search accuracy for SI + text, followed by FI + text and SIs, showing the poorest outcomes when searching for FIs. These results indicated that SI + text had a higher dominant effect on visual search performance of older adults. Such a dominant effect may be partly due to the 3D features of SIs, such as textures, shadows, and highlights to metaphorize real-world objects in appearance and application [15,20], helping older adults to recognize the objects [38]. This dominant effect of SIs on visual searching was also shown by Leung et al. [10]. Additionally, text may help users to better understand the semantic meaning of the icons [22,39]. The combination of icon and text provided redundant information, making it easier for the users to establish substantive connections between new information and relevant anchor concepts in long-term memory and recall the relevant knowledge [40], thereby improving the visual search performance of older adults.

In addition, the visual search performance of all older adults for FIs was not as good as that for SIs in this study. This conclusion was consistent with the findings of Chen et al. [15]. The reason for the difference may be due to the fact that FIs rely on a minimalist visual approach to simplify elements [20], conveying the information to users in too simplistic a manner and ignoring the fact that the human brain is extremely sensitive to visual cues linking graphical user interfaces to the real world [20,41], thereby further affecting the visual search, recognition, and confirmation of icons in older adults with declining memory, attention, perceptual speed, or spatial ability [27,28].

#### 4.1.2. Effects of Cognitive Aging on Icon Search Performance in Older Adults

In the process of icon searching, participants were required to process the visually received feature information of the target icons (e.g., shape, color) and activate their semantic knowledge of icons to correctly associate the features to distinguish the target icons from the distractor (non-target) icons with similar features. As a transitive state between normal aging and dementia, MCI during the incipient stages of the disease may have characteristics of impairments in feature-conjunction processing, similar to Alzheimer’s disease [6]. Patients with MCI have reduced visual attention and control over visual short-term memory, which challenges attention and suppression of irrelevant information while completing search tasks [42,43]. In this study, the older adults with MCI took a longer time to exclude the non-target icons, i.e., took a longer time to search and process the target icons, leading to a longer visual search response time and potentially poorer search accuracy, compared with the older adults in the CN group. This result was consistent with the study by Haesner et al., (2018) on website usability, which found that individuals with MCI took longer time to complete the tasks and generated more errors [44].

#### 4.1.3. Interactive Effect of Icon Types and Cognitive Aging on Visual Search Performance of Older Adults

The results of this study showed that icon types and cognitive aging had interactive effect on the response time, TFF, and FD in the icon search task of older adults. Among the 4 icon types, the participants had the shortest search response time, the fastest search speed, and the fastest processing speed for SI + text. However, these differences in performance between the CN and MCI groups were not significant, suggesting that SI + text may shorten the difference in performance between individuals with MCI and CN and allow older adults to achieve the best search performance compared with the other types of icons. Although Reddy et al., (2020) proposed that combinations of icons and text put more pressure on the limited visuospatial processing resources of older adults, the 3D features and metaphorical methods of SIs can help older adults establish a direct connection between icon content and familiar things in daily life [20], thereby helping the older adults to search and identify icons rapidly. In addition, text and labels are also important for older adults [21]. The addition of text to the icons serves as an explanation to provide addition information to aid comprehension in individuals with MCI, thereby reducing the negative effects of MCI on visual processing speed, memory, language, visuospatial function, and execution.

Regardless of CN or MCI, the visual search performance for FIs in older adults was the worst among the four types of icons, indicating that participants spent a longer time excluding non-target icons from FIs and had a longer cognitive processing time and search response time for FIs. Moreover, the search performance of the MCI group was significantly worse than that of the CN group. FIs with consistent features and unclear metaphors may be especially challenging for users with MCI [20].

### 4.2. Limitations and Future Research

This study has a few limitations. First, this study only included two types of mainstream icons, FIs and SIs, and only performed subjective evaluations on the complexity, familiarity, and semantic distance of the created icons, without eliminating the subjective bias of sampling. Furthermore, all of the icons are taken from the hospital medical system websites and medical public self-service terminal interfaces with the design characteristics of low complexity. As a result, the findings of this study may only apply to icon types with low complexity. Future studies may further investigate the design characteristics of FIs and Sis, which would lead to better understanding of the link between the complexity of icons and visual search behaviors, strategies, and search performance in older adults. Second, although this study has some important findings, it also has a lack of representation due to the small number of participants. A larger size of the sample is needed to further verify our discovery. Third, we used only the icons related to healthcare systems. Thus, the results and analysis of this study are most suited for differentiating the visual search performance of older adults in healthcare scenarios. Using icons with different systems may produce varied outcomes. Future work can use icons from different systems to investigate the impact of icon type and cognitive aging in older adults on visual search performance.

## 5. Conclusions

Although numerous studies have shown that older adults prefer SIs and have better visual performance with SIs, the impact of differences in cognitive aging among older adults remains unclear. Previous studies have only analyzed data such as task completion time and task accuracy for task performance, but the influence of the design of icon types and cognitive aging on the eye movement behavior of icon searching in older adults is still unknown. Therefore, this study used eye-tracking technology to collect the data of the TFF and FD among the participants during the icon search process to analyze their icon search speed and cognitive processing speed.

The following are the study’s findings. First, it was necessary to provide SIs + text for the older adults to assist in the visual search task by increasing the search speed and accuracy. Although there is a trend of transitioning from SI to FI design, FIs should be used with caution in icon design and selection, especially for older users. Second, the older adults with MCI had a significantly poorer performance on the icon search task, compared with the older adults in the CN group. Therefore, MCI factors should be included in GUI design and research related to older adults. Finally, considering the differences in the degrees of cognitive aging among older adults, SI + text should be provided to minimize the search performance differences between individuals with MCI and CN individuals.

## Figures and Tables

**Table 1:** Mean scores and standard deviation for icon complexity, familiarity, and semantic distance.

	Flat Icons (*n* = 27)		Skeuomorphic Icons (*n* = 27)		*F*	*p*
	M	SD	M	SD		
Complexity	2.77	0.08	2.81	0.09	2.78	0.101
Familiarity	3.28	0.06	3.31	0.06	3.53	0.066
Semantic distance	5.76	0.09	5.80	0.11	2.28	0.137

**Table 2:** Demographic information of the older adults in the cognitively normal (CN) group and the mild cognitive impairment (MCI) group.

	NC	MCI
N	8	8
Sex	M = 4; F = 4	M = 4; F = 4
Age (SD ^1^)	62.13 (2.10)	61.25 (1.39)
Education level (SD)	8 (1.41)	8.88 (2.70)

^1^ SD, standard deviation.

**Table 3:** Mean (standard deviation) of visual search accuracy, response time, time to first fixation (TFF), and fixation duration (FD) for different icon types and cognitive levels.

	Accuracy	Response Time (s)	Time to First Fixation (s)	Fixation Duration (s)
Flat icons (FIs)	0.40 (0.04)	23.80 (1.07)	8.05 (0.16)	4.78 (0.06)
Skeuomorphic icons (SIs)	0.74 (0.04)	16.64 (0.28)	5.04 (0.12)	3.53 (0.04)
FI + text	0.95 (0.04)	14.69 (0.41)	3.05 (0.06)	3.18 (0.04)
SI + text	0.96 (0.02)	14.09 (0.29)	2.90 (0.08)	2.95 (0.06)
CN	0.81 (0.03)	15.45 (0.57)	4.37 (0.81)	3.11 (0.05)
MCI	0.71 (0.03)	19.15 (0.53)	5.15 (0.76)	4.11 (0.05)
FI × CN	0.51 (0.16)	20.46 (1.34)	7.41 (0.66)	4.3 (0.30)
FI × MCI	0.28 (0.15)	27.14 (5.47)	8.69 (0.55)	5.27 (0.19)
SI × CN	0.77 (0.14)	14.69 (1.08)	4.47 (0.38)	2.93 (0.20)
SI × MCI	0.70 (0.15)	18.59 (1.06)	5.62 (0.52)	4.13 (0.14)
FI + text × CN	1.00 (0)	13.04 (0.89)	2.85 (0.25)	2.77 (0.22)
FI + text × MCI	0.90 (0.19)	16.34 (1.97)	3.26 (0.21)	3.58 (0.12)
SI + text × CN	0.97 (0.08)	13.63 (0.96)	2.77 (0.42)	2.43 (0.31)
SI + text × MCI	0.95 (0.09)	14.56 (1.24)	3.02 (0.17)	3.47 (0.19)

**Table 4:** ANOVA results for icon type and cognitive level.

	Response Time			Accuracy			Time to First Fixation			Fixation Duration		
	*F*	*p*	*η_p_* ^2^	*F*	*p*	*η_p_* ^2^	*F*	*p*	*η_p_* ^2^	*F*	*p*	*η_p_* ^2^
Icon type	69.56	0.000	0.84	67.08	0.000	0.84	482.00	0.000	0.97	288.59	0.000	0.98
Cognitive level	22.78	0.000	0.64	7.48	0.017	0.37	48.28	0.000	0.79	195.90	0.000	0.94
Icon type × Cognitive level	4.88	0.006	0.27	2.08	0.118	0.14	5.63	0.003	0.30	2.79	0.053	0.18

## Data Availability

Not applicable.
